# How Displaced Migratory Birds Could Use Volatile Atmospheric Compounds to Find Their Migratory Corridor: A Test Using a Particle Dispersion Model

**DOI:** 10.3389/fnbeh.2016.00175

**Published:** 2016-10-17

**Authors:** Kamran Safi, Anna Gagliardo, Martin Wikelski, Bart Kranstauber

**Affiliations:** ^1^Department of Migration and Immuno-Ecology, Max Planck Institute for OrnithologyRadolfzell, Germany; ^2^Department of Biology, University of KonstanzKonstanz, Germany; ^3^Department of Biology, University of PisaPisa, Italy

**Keywords:** particle dispersion model, orientation and navigation, bird migration, homing behavior, atmospheric aerosols

## Abstract

Olfaction represents an important sensory modality for navigation of both homing pigeons and wild birds. Experimental evidence in homing pigeons showed that airborne volatile compounds carried by the winds at the home area are learned in association with wind directions. When displaced, pigeons obtain information on the direction of their displacement using local odors at the release site. Recently, the role of olfactory cues in navigation has been reported also for wild birds during migration. However, the question whether wild birds develop an olfactory navigational map similar to that described in homing pigeons or, alternatively, exploit the distribution of volatile compounds in different manner for reaching the goal is still an open question. Using an interdisciplinary approach, we evaluate the possibilities of reconstructing spatio-temporally explicit aerosol dispersion at large spatial scales using the particle dispersion model FLEXPART. By combining atmospheric information with particle dispersion models, atmospheric scientists predict the dispersion of pollutants for example, after nuclear fallouts or volcanic eruptions or wildfires, or in retrospect reconstruct the origin of emissions such as aerosols. Using simple assumptions, we reconstructed the putative origin of aerosols traveling to the location of migrating birds. We use the model to test whether the putative odor plume could have originated from an important stopover site. If the migrating birds knew this site and the associated plume from previous journeys, the odor could contribute to the reorientation towards the migratory corridor, as suggested for the model scenario in displaced Lesser black-backed gulls migrating from Northern Europe into Africa.

## Introduction

The use of olfactory cues for chemotaxis and navigation is well known and widespread in insects (Vickers, 2000; Reinhard et al., 2004; Jacobs, 2012) and other taxonomic groups (Wiener et al., 2011), but has until recently been controversial in birds (Wallraff, 2003, 2015; Alerstam, 2006). The avian olfactory navigation hypothesis was proposed by Papi in the early 1970s to explain the experimental evidence that: (a) homing pigeon develop unimpaired navigational abilities only if they are exposed to the natural winds at the home area (Wallraff, 1970a,b); and (b) anosmic homing pigeons are dramatically impaired at homing (Papi et al., 1972). According to this hypothesis the navigation mechanism of homing pigeons is composed of two phases: (i) a learning phase in which homing pigeons learn the association between the wind borne odors and the direction of the winds blowing at the home area (Ioalè et al., 1990); and (ii) an operative phase in which the displaced birds determine the direction of displacement by recognizing the release site prevalent local odors and recalling the direction these odors came from at the home area (Papi et al., 1973; Wallraff, 1990).

While a large body of experimental evidence in support of olfactory navigation (Gagliardo et al., 2006) and the specific role of environmental odors in homing pigeons navigation accumulated (Benvenuti et al., 1973; Wallraff, 2005; Gagliardo et al., 2011a,b; Gagliardo, 2013), little is known about the distribution of the volatile compounds and even less about the kind of molecules potentially used in the olfactory map (Waldvogel, 1987; Wallraff, 1989, 2013). However, Wallraff and Andreae (2000) conducted a chromatographic study on samples of air collected within a wide region around a pigeon loft used for many navigational experiments, and showed that volatile organic compounds were distributed along fairly stable gradients. In addition, a simulation test showed that stable ratios of at least three different volatile compounds seemed to provide sufficient information for allowing a homeward orientation of “virtual” pigeons, whose behavior was comparable to that observed in real birds (Wallraff, 2000).

A paramount question is whether olfactory navigation is a unique feature of homing pigeons or whether it is a widespread mechanism in birds (Wallraff, 2004). Some encouraging results in support of a common use of olfaction in avian navigation (Fiaschi et al., 1974; Wallraff et al., 1995) have recently been accumulated by tracking studies reporting impaired navigation abilities in anosmic shearwaters displaced far from their nesting colony (Gagliardo et al., 2013; Pollonara et al., 2015) and in two species of migrating birds made anosmic and displaced far from their migratory corridor (catbirds, Holland et al., 2009; black backed gull Wikelski et al., 2015). The use of environmental olfactory information for navigation in wild avian species opened a number of questions on the way olfactory signals might be learned and exploited in general by migratory birds. For example, young shearwaters might learn an olfactory map during their time on, and explorative flights around, the native colony before their first migration, in a way similar to that described for homing pigeons (Waldvogel et al., 1978), i.e., by associating odors with wind directions. However, since shearwaters use olfaction not only to locate their own nest within their colony, but also for finding their foraging sites in the open ocean (Grubb, 1974; Nevitt et al., 2008; Reynolds et al., 2015), they might learn the differential distribution of biogenic odors characterizing different sea areas while wandering across the oceans for thousands of kilometers during the non-reproductive period. In this way they might learn an olfactory landscape of the visited oceanic areas—assuming there exists some consistency in atmospheric odors over the sea (Nevitt and Bonadonna, 2005)—in a similar way terrestrial birds learn visual topographical cues of the areas they flew over.

Wikelski et al. (2015) reported for the first time a GPS tracking study supporting olfactory navigation in migrating Lesser black-backed gulls belonging to a population breeding in Finland and displaced to Heligoland, outside their familiar range. Birds with an intact olfactory apparatus sooner or later joined the migratory corridor of their population from the places they were translocated to, eventually reaching their wintering sites (Nile delta or Lake Victoria). The birds subjected to a section of the olfactory nerves headed straight South instead, ending their journey far from the migratory target, in an area not normally used as wintering or non-breeding grounds in this species. How migrating birds such as Lesser black-backed gulls could have learned and exploited environmental odors for finding back to their migratory corridor is presently unknown. The birds might have learned an olfactory map by associating the wind direction with the odors carried by the winds at each stopover site, as hypothesized by Wallraff (2005). In this view the stopover sites would not be olfactory signposts, and the birds would have a more or less extended olfactory map at each stopover site, depending on the information carried by the winds blowing at the stopover site during the stopover period. Under this scenario, if displaced from their migratory corridor, the birds would determine the direction of displacement on the basis of local odors at the release site. A second hypothesis, that does not necessarily exclude the previous one, is that migratory birds might have learned characteristic odors of some stopover sites and after displacement, might have been helped by a plume transporting particles from these stopover site to the bird’s current location, by simply flying against the plume. Under this scenario, the plume would not have to be permanently available, but instead it could be sufficient to get a “nose of air” from approximately the right direction once in a while. In this case, each stopover site would act like an olfactory signpost. It should be noted, however, that the use of the plume is not an olfactory navigation in a strict sense (it does not imply the use of an olfactory map), but rather chemotaxis, i.e., orientation towards or away from an olfactory source. This mechanism of using environmental odors is much simpler compared to an olfactory map, and does not require high cognitive abilities. However, reaching a goal simply by flying against a plume can be done only under certain wind conditions where particles originating from the goal can reach the subject. Nevertheless, it might have other advantages, such as a fast and simple learning.

So far the use of an olfactory plume was not considered necessary in birds and remained undemonstrated. For example, intact shearwaters displaced east from the Azores were able to find their way home despite the fact that the winds were not carrying the home island information (the winds were never blowing from west during the birds’ homing flight; Gagliardo et al., 2013). Similarly, in homing pigeons many experiments speak against the use of a plume, as homing pigeons were shown to head home even if released in anosmic condition, provided that they had been exposed to the olfactory information of the release site prior to the release (Wallraff, 2005; Gagliardo, 2013; Gagliardo et al., 2016). Although it seems unlikely that olfactory signals-based navigation might be exclusively based on chemotaxis, the ability of migrating birds to take advantage of wind borne odors originating from particularly important stopover cannot be excluded *a priori*.

Increasing computational capacities and recent innovations in modeling and the reconstruction of atmospheric flow are currently providing weather data at continental to global scale at increasingly higher spatial and temporal resolution. Atmospheric scientists are increasingly able to redraw the purported chemical trails and the estimated path of particles in the atmospheric column with the help of particle dispersion models in a spatially and temporally explicit way with increasing precision and accuracy (Stohl et al., 1998, 2005; Hegarty et al., 2013).

Here, we use the commonly used model FLEXPART^1^ (Stohl et al., 2005) to evaluate a particle dispersion model in conjunction with wind data obtained from the European Centre for Midrange Weather Forecast (ECMWF^2^). We attempt to reconstruct the origins of particles arriving at specific locations of 20 trans-located and sensory manipulated Lesser black-backed gulls (*Larus fuscus*) equipped with GPS devices (Wikelski et al., 2015), using strong simplifying assumptions. FLEXPART allows the reconstruction of the origin of emission of particles with respect to a specific location backwards in time, a feature used to locate the potential source of pollutants arriving at specific places (Seibert and Frank, 2004). Here, we use this tool to assess the extent to which a plume originating from the migratory corridor of Lesser black-backed gulls migrating from Finland, could have affected the orientation of birds that were either able or unable to smell.

## Materials and Methods

**Figure 1:**
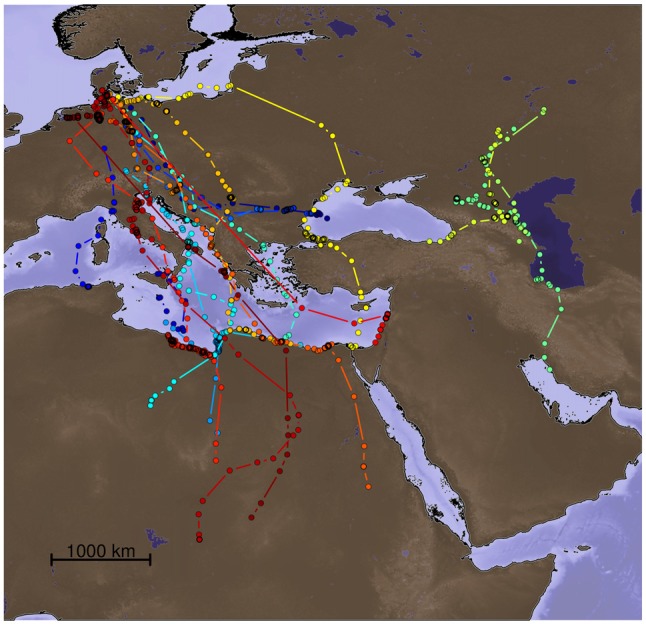
**Migratory sections of the tracks of the 20 Lesser black-backed gulls (*Larus fuscus*) for which particle dispersion models were calculated.** The data are from Wikelski et al. (2015). Each color represents a different individual.

We modeled particle dispersion for migratory trajectories of 20 Lesser black-backed gulls (*Larus fuscus*) used in a recent experimental study investigating the role of different sensory modalities for true navigation during migration (Figure 1; Wikelski et al., 2015; data repository: doi:10./001/1.q986rc29). The tracks for which we calculated the particle dispersion models were: (i) birds with their olfactory nerves sectioned (ONS); and (ii) gulls with intact olfactory nerves (ION). The latter group was composed of both un-manipulated gulls and gulls subjected to the section of the ophthalmic branch of their trigeminal nerves. The displaced birds were trans-located from their breeding grounds to presumably unfamiliar areas between 850 and 1000 km West and East, respectively (for details see Wikelski et al., 2015).

For the time between the start of the migration and when each individual reached the coast of North Africa, we linearly interpolated the locations to hourly estimated positions on a straight line connection between the known positions for each individual. For each of these locations and times, including the GPS reported locations, we modeled the origin of particles that arrive. To model the particle dispersion, we made a few simplifying assumptions. First, chemicals carried by winds were assumed to be aerosols. Underlying this assumption is a series of physical attributes that result into a certain decay and propagation in the atmospheric column, in turn defining the FLEXPART model outcome. In addition, we assumed, due to the lack of alternative realistic scenarios, that massless aerosols were emitted at a spatially uniform and constant rate throughout the entire area (Northern Europe to the north coast of Africa). The emission rate refers to how many presumed particles a specific landscape emits into the atmospheric column per unit of time. We further allowed aerosol particles to travel a maximum of 3 days (SOM V1). Thus, for any known location of the birds, using the above mentioned assumptions, we assessed the average putative concentration of aerosols that could potentially have been received by the bird and traced them back to where they were emitted. We used an air-tracer to reconstruct the movement of air masses with three different specified upper boundaries. Based on a global output grid of a 0.2° resolution, we simulated three atmospheric levels with an upper boundary at 100, 3000 and 50,000 m. Using a backward simulation with each gulls’ location and time as the target location, including the interpolated hourly locations, we assumed that the gulls had a 5 min reception period at the respective locations and times, and that the perceived particles arriving at the locations during the specified times could be detected from 0 m to 30 m above ground level. For each simulation 100,000 particles were emitted in total over the entire area in question defined by the 3 days travel time allowed for the particles. Once the simulations using FLEXPART were concluded, we converted the output data with the python library “pflexible” (Burkhart, 2011) to R rasters and summed the results of the simulations across altitudes as well as the entire time period to reconstruct the relative density of paths of particles arriving at the gull locations.

Chemotaxis has received little support in the avian olfactory navigation theory because under the simplest scenario, it requires high temporal stability of the olfactory information, thereby allowing the birds to follow the traces to the source for as long as the information is available. Using the particle dispersion model we looked at whether, as a prerequisite for gradient climbing, the olfactory information was persistent enough to enable the birds to follow the olfactory information over the periods of the time necessary to trace back to their migratory corridor. If translocated birds were able to trace their way back, we expected the spatial pattern in the concentration gradients of the particles received at the birds’ locations to autocorrelate, and this correlation should be maintained over time. We therefore calculated a correlogram which quantifies the correlation coefficient between subsequent FLEXPART model outputs as a function of time between them.

In addition, we calculated how many particles the birds belonging to the ION and SON groups could have “collected” over the entire course of their migratory journey. We expected that, if olfactory information is used continuously and repeatedly throughout the journey, the anosmic birds would be less likely to follow olfactory traces and thus collect more particles along their migratory journey than the birds capable of smelling, as the latter react to the olfactory information and thus climb olfactory gradients quickly. Thus, anosmic birds should be exposed to a lot of olfactory information from their migratory corridor and not react to it, whereas control birds should use little olfactory information to return to their migratory corridor quickly. We therefore calculated, for each hourly raster, the average putative particle densities potentially perceived at the gull’s position. We then summed the particle density values for any given time step for all the locations the bird visited. In short, using this approach we calculated the particles that a bird would collect after having received the olfactory information and potentially reacting to it by changing its path and subsequently tracking the odor particles to the origin of their releasing sites. Under this scenario, a bird does not necessarily climb a concentration gradient and the results are therefore not affected by the presence or absence of a potential olfactory plume. To account for individual differences in migratory speed and length of trajectories, we calculated the total sum of the accumulated particles divided by the number of locations for each bird.

Finally, we determined for 17 of the 20 Lesser black-backed gulls in a binary classification whether, or not, an individual got aerial information from either the original migratory corridor or the goal areas (see Wikelski et al., 2015). Subsequently, we determined whether a bird changed its flight direction towards these areas over the course of 500 km before and 500 km after this potential information was received. For this analysis, we left out the three individuals migrating along the Caspian sea because their regular migratory corridor was unknown. We thus investigated whether the birds could have followed a chemical cue, they possibly associated with a location they wanted to reach.

Using these two analyses, we investigated whether the birds, after perceiving a transient olfactory information, reoriented and subsequently moved towards the migration corridor from which they were displaced. In essence, we modeled the path of all particles arriving at the birds location from all locations over the entire tracking period. For visual clarity, we restrict our figures to a certain level of aerosol concentrations, representing the paths of particles arriving at each birds’ locations at an hourly interval (for a window of 5 min) while being emitted over continental Europe during the past 3 days prior to each hourly location of each of the birds.

## Results

**Figure 2:**
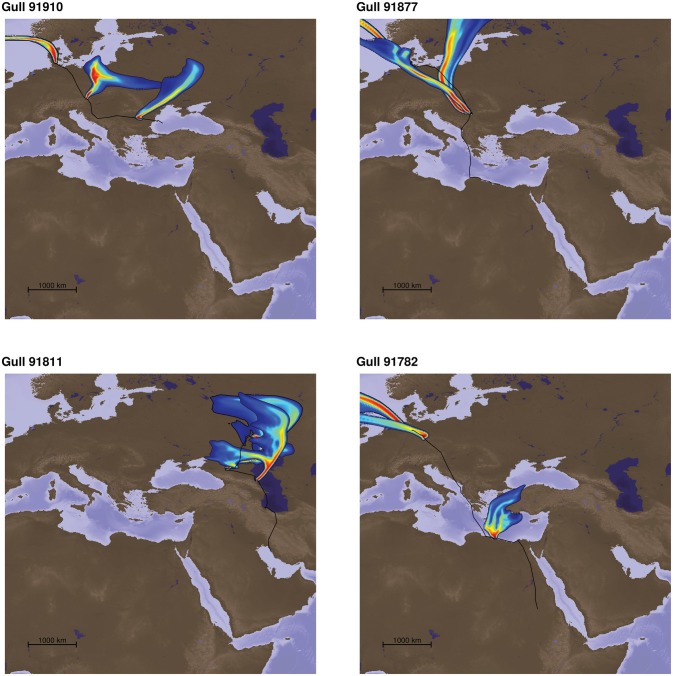
**Four examples of odor plumes representing the source location of the particles arriving at three positions of each individual track (start, position at 1/3 an 2/3 of the total track).** The colors represent the relative contribution of the sources to the particle mixture modeled to arrive at each respective position, with warm colors representing higher contributions. These particle dispersion models were calculated for all hourly interpolated locations of all 20 bird tracks used in the study. The individual gull tracks from data repository doi:10./001/1.q986rc29. We could include in our study were 91910, 91750, 91745, 91911, 91908, 91907, 91864, 91821, 91823, 91811, 91845, 91881, 91819, 91877, 91852, 91782, 91871, 91916, 91802, 91783. The three gulls left out for the corridor analysis were 91811, 91845, 91881. Please see Supplementary Material for videos showing odor plumes.

We calculated 12,939 hourly particle clouds using FLEXPART for 20 birds along their movement trajectories. Aerial information proved highly dynamic as a consequence of the atmospheric conditions during the migratory period. Figure 2 exemplifies the plumes perceived by individual gulls at three different locations during their migratory journeys (at the beginning, 1/3 and 2/3 of the track). In general, it can be said that particles arriving at the birds’ locations had a temporally highly varying spatial provenance, yet we found little evidence for turbidity or stochasticity, i.e., there was no formation of random islands of particle sources or strongly deflected distant sources of aerosols that could not be associated with wind direction for orientation. Thus, the origins of the particles were usually spatially highly concentrated and the aerosol particle clouds were funneled, showing clear directional characteristics (Figure 2 for example; see also Supplementary Videos in “Supplementary Presentation 1”).

**Figure 3:**
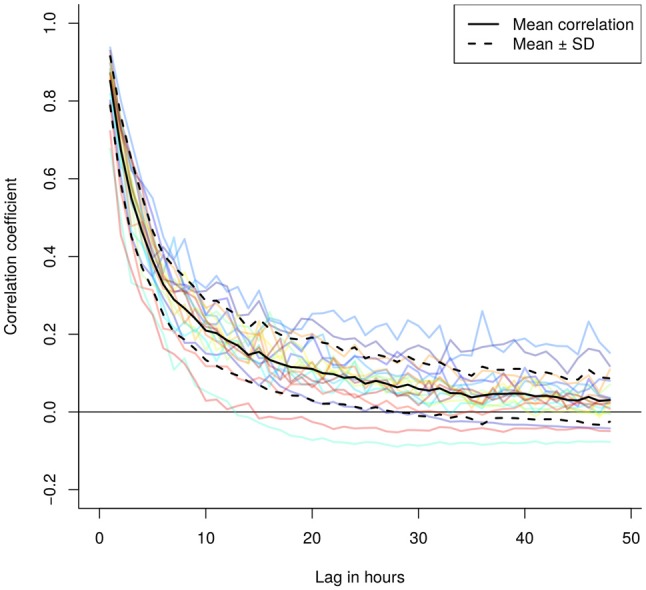
**Correlogramm of the hourly and spatially explicit particle dispersion models showing a steep decrease in correlation between modeled particle dispersion models over time.** The lag indicates the difference in hours between two models, where we selected for lags between 1 and 48 h for each 50 (if present) random pairs of model predictions to calculate the average individual correlation of model predictions as a function of the time lag (colored lines). The black solid and dashed lines represent the mean ± standard deviation.

The correlogram (Figure 3), expressing the overlap between subsequent combinations of the spatially explicit model predictions up to 48 h within the same individual, suggested very low similarity at low temporal lags, further decreasing rapidly. After on average 24 h the correlation between the concentrations dropped to almost zero.

**Figure 4:**
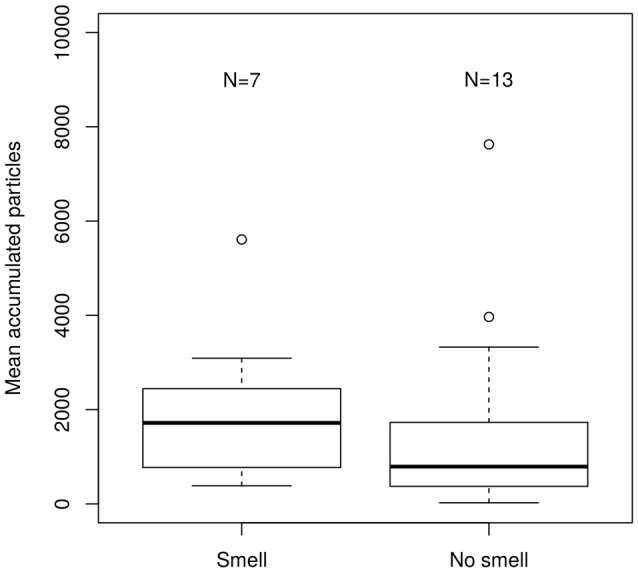
**Boxplot of the accumulated particles for two treatment groups.** “Smell” the birds capable of smelling and “No smell” those incapable of smelling. The values represent the source contributions as modeled by FLEXPART by spatially overlaying the predicted particle dispersion model output with the future locations of positions where the birds were considered having actively moved to by applying a velocity threshold of 2 ms^−1^ between two consecutive locations. The difference in the two classes of birds was non-significant according to Wilcoxon rank-sum test (*p* > 0.05).

The comparison of the accumulated particles during the migratory period suggested a trend towards lower particle accumulation in the birds capable of perceiving olfactory information vs. the anosmic birds with their sectioned olfactory nerves (Figure 4). The control and the trigeminal sectioned birds showed similar levels of accumulation as the anosmic birds.

Out of the 17 individuals migrating South after being dislocated to Heligoland from their original Finnish population, 11 had an intact sense of smell and six were deprived of it. Eight out of these 11 individuals received particles from the population’s migratory corridor and reoriented and joined the original migratory corridor. The remaining three did not receive particles, according to the FLEXPART models, of which two did not join the migratory corridor, whereas one eventually did. This one gull apparently followed the coastline of North Africa visually and in this way ended up within the migratory corridor.

For eight birds for which we had detailed GPS information for 500 km before and 200 km after they changed their direction of travel, we observed that they significantly changed their orientation after having been exposed to the aerial information coming from the migratory corridor (Hotelling test for paired data, *F* = 204, *P* < 0.001), and significantly oriented towards the plume direction (birds’ distribution setting the plume direction to 360°; mean vector length and direction: *r* = 0.75, α = 356°, V test *P* < 0.001). Therefore the exposure to the plume from the migratory corridor seemed to have significantly affected their orientation producing an important change of direction towards the goal.

**Figure 5:**
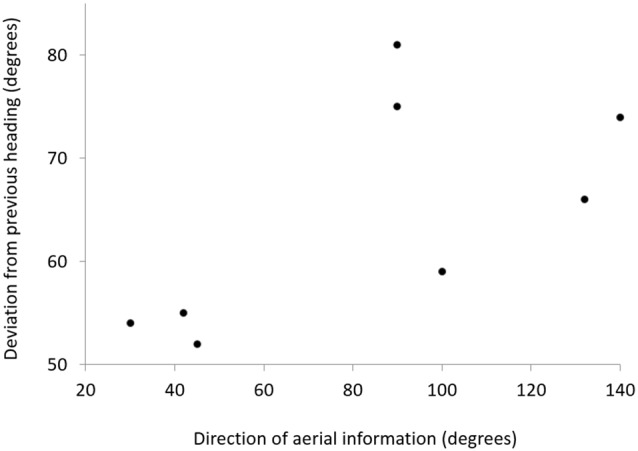
**Plot showing the relationship between the direction of aerial information relative to the flight direction of the gull over the last 500 km and the change in flight direction for the next 200 km after receiving aerial information from the migratory corridor.** Each point represents one gull. The direction of the plume was determined over the last 30 km before being perceived by the bird.

For the eight birds that reoriented, the directional change in the flight path towards the migratory corridor was positively correlated with the angle at which the aerial information arrived at the bird, i.e., the wind direction over the last 30 km before the wind arrived at the location of the bird. If aerial information, i.e., particles carried by winds from the migratory corridor, arrived at the bird as a tailwind (i.e., from NNE during the southward migration), the bird made only a small correction towards the migratory corridor (i.e., towards the SSE; Figure 5). If the aerial information from the migratory corridor arrived more as a headwind (i.e., from ESE), the bird corrected it’s path at a steeper angle towards the migratory corridor.

Of the six gulls without a sense of smell, all received particles and only one bird apparently reoriented, i.e., changed its direction between the previous 500 km and 500 km after receiving particles from the migratory corridor. A Chi-square test suggested that the pattern of reorientation depending on the ability to smell or not was not random (*X*^2^ = 13.5, *n* = 17, *P* = 0.025).

## Discussion

We used a particle dispersion model in combination with the tracking data of migratory Lesser black-backed gulls to gain first insights and testable hypotheses for the future as to how aerial information could be used by migratory birds when displaced from their regular migration corridor. The particle dispersion model allowed us to get a first estimate on the possible origins of the particles arriving at the birds’ locations.

The analysis of the behavior of the birds reorienting in the expected direction suggests that despite the admittedly naïve models we used, the model outcome could have biological relevance. Although we cannot exclude the use of an olfactory map, our analysis suggested that birds with an intact olfactory apparatus might have exploited information borne by a air plume originating from the migratory corridor. The birds showed a significant change in direction after having been exposed to the wind from the direction of the migratory corridor. However, the extent of their deflection might depend on the angle at with their received their purported aerial information from the migratory corridor. With tail wind from the migratory corridor, the birds corrected with small angular changes towards the corridor, because flying against wind would be incompatible with their migratory direction. Whenever they received lateral or headwinds from the migratory corridor, the birds reoriented more steeply towards the corridor. Such a behavior could indicate a simple “rule of thumb” that would make sense under most atmospheric conditions, i.e., “adjust the angle of reorientation according to the direction you receive the wind that carries the particles originating from the migratory corridor” (Figure 5). The use of a plume carrying chemical cues originating from the goal was hypothesized for white-chinned petrel displaced at relatively short distance from the island (Benhamou et al., 2003). Here we report for the first time, a case of long distance displacement for which the wind pattern observed during tracking experiments are not incompatible with the use of a plume.

A reorientation based on information carried by a plume originated from the migratory corridor implies that the experienced (adult) birds must have previously been exposed to, and should have memorized, at least some of the specific local odors along their original migratory route. Therefore, both for the use of a plume and for the use of an olfactory map, a high level of fidelity to the same migratory corridor through successive migratory flights might enrich and strengthen olfactory learning, depending of the level of stability of the odor patterns through different years.

Applying the particle dispersion models also revealed and identified the parameters that could largely affect the model outcome. However, a more realistic approximation of the origin and concentration of aerosols received at the location of the birds requires not only detailed knowledge of emission rates, but also knowledge about the particle chemistry and sensitivity of the animals towards different chemicals at neuro-receptor levels for each individual. Such information is currently not available, but can be gathered in the future.

The prediction quality of FLEXPART is essentially influenced by the accuracy of the underlying tracer algorithms and their limitations, but also strongly influenced by the assumptions about the aerosol chemistry (Stohl et al., 1998; Hegarty et al., 2013). Applying a particle dispersion model therefore revealed some gaps in our knowledge that could be addressed in the quest for more realistic predictions. The chemical compounds are the most important and essential factor unknown in the quest to a more realistic modeling of the olfactory information perceived. Although we know that birds use olfactory information, next to nothing is actually known about the identity of the molecules involved, their persistence in the environment, and the rates with which they are produced and emitted (Wallraff and Andreae, 2000). We arbitrarily chose default physical attributes of aerosols under the assumption that the olfactory chemicals should at least be partially water soluble and therefore accumulate and propagate like aerosols. But knowing more about the chemicals and their fate when emitted would clearly help to better predict their concentration, their halftime and how they decay into other chemicals, which in turn potentially can have additional, altered information content, for example, about distance and thus reliability of the association of wind direction and olfactory target. Next, it would be highly informative to know more about the identity and the physical attributes of the particle sources, the chemicals suspected to be involved in the navigation and orientation behavior. At the receiver, i.e., the bird’s brain, it would be beneficial to know the behaviorally relevant perception thresholds for the various chemical suspects in the cocktail of chemical compounds received and used for orientation. Finally, another big unknown in the communication chain is the emission rate with which the relevant chemicals are produced and emitted. Large-scale *in situ* measurements of emission rates will probably prove unrealistic, but once the chemicals are identified, it should be possible to find proxies that can be measured, ideally using remote sensing tools to extrapolate likely release rates at the relevant spatio-temporal scales. Insight into these chemicals and their distribution can also inform experiments where, for example, *a priori* predications can be made for decisions of migrants based on dispersion models and knowledge of release rates.

Finally, the model predictions should ideally be verified to become further improved following a reanalysis approach (Stohl et al., 1998). Both particle dispersion models as well as weather models have limitations that affect the prediction outcomes and should be improved. In large scale weather models, local wind patterns cannot be well reflected and maybe one of the major bottlenecks in applying FLEXPART. We suggest the combination of regional atmospheric models such as RAMS (Pielke et al., 1992) and FLEXPART could yield acceptable high resolution dispersion models. A procedure commonly adopted in spatial statistics, where model predictions are re-analyzed after a first round of analysis, by re-fitting the predictions to independent measurements could be a way forward, if independent measurements were available. Novel tools might help, as it is becoming feasible to measure chemical composition of the particles *in situ*, which can provide the independent measurements required for such re-analysis models. From the perspective of the neurobiology of olfactory navigation, the combination of such aerial chemical sampling while simultaneously recording the electro encephalograms and olfactory nerve activity of free-flying birds could soon yield insights into candidate chemicals acting as messengers in olfactory navigation (Vyssotski et al., 2009).

A wealth of studies exist that have investigated chemotaxis and olfactory navigation along pheromone trails on substrate and aerial particles in non-avian species, mainly in insects and mammals and often under controlled laboratory conditions (summarized, e.g., in Wiener et al., 2011). The understanding of the different factors influencing olfactory navigation and the use of wind-borne odor cues is not only important to navigation in birds, but for a wide range of animals with possibly broad evolutionary implications. The olfactory spatial hypothesis (Jacobs, 2012) postulates that olfactory maps influence the brain organization in the animal kingdom, more specifically the olfactory bulb and hippocampus in vertebrates (Jacobs, 2012). Modeling particle dispersion for other taxa could therefore provide an additional tool to answer some questions, particularly in species where more is known about the olfactory system than in birds. In frugivorous and nectarivorous bats (*Phyllostomidae* and *Pteropodidae*), for example, it has been shown that sulfurous compounds produced by the host plants may act as olfactory cues (Dechmann and Safi, 2005). In these systems, emission rates could be measured and/or manipulated more easily. Comparative studies suggested that in these bats the different tasks of navigation over long and short distance have selected for the combination of large olfactory bulbs and hippocampi relative to body sizes (Safi and Dechmann, 2005; Dechmann and Safi, 2009). However, although the chemical compounds in various study systems are known, we fear that the complexities involved in reconstructing the olfactory landscapes are much more challenging than for birds at the scale that we presented here. Modeling particle dispersion in dense forests, for the bat example, or in the wild at a much smaller spatial scale of a few hundred meters, for insects and other small scale studies, seems like a challenging task, due to the effect of turbulences at those scales. Nevertheless, such studies have successfully been conducted (e.g., Vickers, 2000; Reinhard et al., 2004; Gaudry et al., 2012).

In the future, bringing together atmospheric science, movement ecology as well as neuroscience and chemical ecology while combining laboratory-based experimental approaches with data collection and studies at the relevant landscape levels may prove beneficial for all involved disciplines, and the question how animals navigate globally in their physical environment. Particle dispersion models, when refined and improved in the way we suggest above, may provide helpful tools in understanding and predicting the environmental conditions relevant for many biological processes happening in the highly dynamic atmospheric column.

## Author Contributions

KS, MW and BK jointly conceived the study and analyzed the data. All authors wrote the manuscript.

## Funding

Funding was provided by the Max Planck Institute for Ornithology.

## Conflict of Interest Statement

The authors declare that the research was conducted in the absence of any commercial or financial relationships that could be construed as a potential conflict of interest.

## Footnotes

^1^https://www.flexpart.eu/

^2^http://www.ecmwf.int/
